# Application of Response Surface Method for Preparation, Optimization, and Characterization of Nicotinamide Loaded Solid Lipid Nanoparticles

**DOI:** 10.15171/apb.2018.029

**Published:** 2018-06-19

**Authors:** Molood Alsadat Vakilinezhad, Shima Tanha, Hashem Montaseri, Rassoul Dinarvand, Amir Azadi, Hamid Akbari Javar

**Affiliations:** ^1^Department of Pharmaceutics, Faculty of Pharmacy, Tehran University of Medical Sciences, Tehran, Iran.; ^2^Department of Quality Control, Faculty of Pharmacy, Shiraz University of Medical Sciences, Shiraz, Iran.; ^3^Nanotechnology Research Centre, Faculty of Pharmacy, Tehran University of Medical Sciences, Tehran, Iran.; ^4^Department of Pharmaceutics, School of Pharmacy, Shiraz University of Medical Sciences, Shiraz, Iran.

**Keywords:** Nicotinamide, Response surface method, SLN, Stearic acid

## Abstract

***Purpose:*** Solid lipid nanoparticles (SLNs) have been proven to possess pharmaceutical advantages. They have the ability to deliver hydrophilic drugs through lipid membranes of the body. However, the loading of such drugs into SLNs is challenging. Hydrophilic nicotinamide, a histone deacetylase inhibitor, is used to establish SLNs with enhanced encapsulation efficiency by using statistical design.

***Methods:*** The possible effective parameters of these particles’ characteristics were determined using pre-formulation studies and preliminary tests. Afterwards, the Response Surface Method (RSM) was utilized to optimize the preparation condition of SLNs. The effect of the amount of lipid, drug, surfactant, and the mixing apparatus were studied on particle size, zeta potential, and encapsulation efficiency of the obtained particles. The acquired particles were characterized in respect of their morphology, in vitro release profile, and cytotoxicity.

***Results:*** According to this study, all the dependant variables could be fitted into quadratic models. Particles of 107 nm with zeta potential of about -40.9 and encapsulation efficiency of about 36% were obtained under optimized preparation conditions; i.e. with stearic acid to phospholipon® 90G ratio of 7.5 and nicotinamide to sodium taurocholate ratio of 14.74 using probe sonication. The validation test confirmed the model’s suitability. The release profile demonstrated the controlled release profile following the initial burst release. Neither the nicotinamide nor the SLNs showed toxicity under the evaluated concentrations.

***Conclusion:*** The acquired results suggested the suitability of the model for designing the delivery system with a highly encapsulated water soluble drug for controlling its delivery.

## Introduction

Preparation of a controlled release drug delivery system for incorporation of hydrophilic drugs is a concern in the pharmaceutical field. Various investigations have been carried out for this purpose.^1,2^ Solid lipid nanoparticles (SLNs) have attracted considerable attention in recent years due to their advantageous characteristics.^3-5^ Their advantages include biocompatibility, safety, stability, ability to be control-released, and ease of large scale production.^6-8^ SLNs have the ability of delivering hydrophilic drugs through lipid membranes of the body. However, the loading efficiency of hydrophilic drugs is negatively impacted due to their rapid dissolving within the aqueous phase.^9,10^

Nicotinamide (NA) has an aqueous solubility of 691-1000 mg/ml.^11^ Considering its hydrophilic characteristics, it can benefit from the advantages of a controlled delivery system such as SLNs.

NA, the active form of niacin, is a histone deacetylase (HDAC) inhibitor. Histone acetylation plays an important role in chromatin condensation and gene expression. HDACs are among the key responsible enzymes characterizing cell fate. Some diseases such as cancer and neurodegenerative disorders are considered to be the consequence of deregulation in activity of HDACs.^12,13^ In the field of neurophysiology, this deregulation leads to impaired learning and memory.^14,15^ In recent years, NA has attracted scientists’ attention for delaying the progression of Alzheimer’s disease in preclinical studies.^16^ Acquiring the high encapsulated system of this freely soluble compound not only benefits the controlled delivery of NA itself, but can also enunciate the possibility of delivering other hydrophilic drugs.

Numerous parameters affect the properties of a delivery system. The type of lipid used for preparation of SLNs can affect almost all characteristics of the particles, including the particle size and the drug encapsulation efficiency.^17,18^ Various lipids had been evaluated for SLNs preparation. Of all the natural lipids that can be used, stearic acid (SA) and glyceryl monostearate (GMS) were among the most investigated ones. Given their chemical structures, it can be expected that the hydroxyl groups of GMS can undergo interaction with the amide group of NA. Furthermore, it can be expected that SLNs incorporating SA represent appropriate particle size and stability characteristics.^5,19^

Beside the lipid, there are various factors that affect the characteristics of SLNs. These include the concentrations and ratio of the polymer, drug, and surfactant, as well as the preparation situation, including the type of apparatus, mixing intensity, and mixing time.^20^ In fact, providing an optimum preparation condition is a basic step in preparation of any particulate system.

In traditional methods for multi-factor optimization, the effect of one factor is studied at a time while the other factors remain constant. This requires numerous trials which cost a lot of time and expense. Moreover, since in this method the possible interactions between studied factors are not considered, several failures in prediction of the optimum situation can be observed. Mathematical modeling by providing rapid optimization of various influential variables could be considered as a useful replacement for this traditional method.^21-23^

The response surface method (RSM) was used commonly for optimizing the preparation condition, as well as predetermining the properties of the pharmaceutical preparation.^24,25^ The purpose of this project is to design a SLN system with improved encapsulation efficiency of the hydrophilic drug using NA. The effect of different factors, including the type of lipid, the amount of drug and surfactants, and mixing situation were studied on particle size, size SPAN, zeta potential, and encapsulation efficiency of the obtained nanoparticles. Response surface test with D-optimal design was carried out to obtain nanoparticles with appropriate characteristics. The acquired SLNs were evaluated in respect of their morphology, drug release pattern, and cytotoxicity.

## Materials and Methods

Stearic acid was provided from Merck, Germany. Phospholipon^®^ 90G was purchased from Lipoid, Germany. The SH-SY5Y cell line was received from Pasteur institute, Iran; and essential cell culture media components were provided from Gibco, USA. The 3-(4,5-Dimethylthiazol-2-yl)-2,5-diphenyltetrazolium bromide, MTT, was purchased from Sigma-Aldrich, USA. All the others synthetic materials were provided in analytical grade from Sigma-Aldrich, Germany. Required solvents were procured from local suppliers.

### Pre-formulation studies 

Standard curves of NA were prepared in distilled water and n-octanol using UV spectrophotometric method in wavelength of 254 nm.

Solubility and partition coefficient of NA were determined using Organization for Economic Co-operation and Development Test Guideline 105 and 107 (OECD TG 105 and 107), respectively.^26,27^

Partitioning of NA in different lipids were determined (Table 1). Briefly, the stock solution of NA in distilled water was prepared. Tubes containing constant amount of melted lipid, i.e. SA or GMS, or their combinations in different ratio, were prepared separately. Known volume of stock was added to each tube. Tubes were shaken at almost 10 °C above the melting point of the containing lipid. Samples were then cooled and phases were separated by centrifuge. Both aqueous and lipid phases were analyzed for the NA amount contained therein. Lipid partition coefficient was determined using following equation:

$$Pc\;\left(\frac{SA\;and/or\;GMS}{water}\right)=\frac{Concentration\;of\;NA\;in\;SA\;and/or\;GMS}{Concentration\;of\;NA\;in\;water}$$

Possible interaction between NA and lipid(s) was determined by Fourier Transform Infra-Red Spectroscopy (FTIR) studies (VERTEX70 spectrophotometer, Bruker^®^, Germany). Samples were prepared as physical mixtures (NA mixed with lipid(s) at room temperature) and melted-cooled mixtures (NA mixed with melted lipid(s) and then cooled to room temperature). The spectrum of NA, lipids, their physical mixture (ratio of 1:1), and their melted-cooled mixture (ratio of 1:1) were obtained. All spectrums were conducted in range of 400-4000 cm^-1^ and KBr was used for preparation of samples.

SLNs were prepared by microemulsification method using the top two lipid(s) mixtures with higher NA partitioning; i.e. SA, and mixture of SA and GMS with ratio of 2:1. The obtained particles were characterized in respect of their particle size, size SPAN and stability. The better lipid composition was chosen for the further evaluations.

### Preparation of SLNs 

SLNs were prepared using microemulsification method.^28^ SA and phospholipon^®^ 90G were used as the internal oil phase. The external water phase were composed of sodium taurocholate and ultrapure water. The oil phase was heated up to 70 ± 2 °C on a water bath with continues stirring. The water phase was also heated up to the same temperature separately. By adding the oil to the water phase under mixing situation, the primary oil-in-water microemulsion (O/W) was obtained. The microemulsion was dispersed in a cold ultrapure water and mixed for 5minutes. The resulted aqueous dispersion of SLNs were filtered through Whatman^®^ Puradisc Syringe Filter (0.2 µm), washed using cellulose dialysis bag (MWCO 12 KDa) and then lyophilized. To prepare drug loaded SLNs, the aforementioned procedure was performed while NA was dispersed in the melted oil phase.

**Table 1:** Solubility, partition coefficient and lipid partitioning of nicotinamide.

**Solubility**				
		NA in water^(mg/ml)^	NA in n-octanol ^(mg/ml)^	
Preliminary test		714.28	4.347	
Main test		735.10	4.61	
**Partition Coefficient**				
n-octanol:water		NA in water	NA in n-octanol	Partition coefficient
1:1	Test1	0.679	0.291	0.43
	Test2	0.692	0.277	0.40
1:2	Test1	0.787	0.370	0.47
	Test2	0.791	0.387	0.49
2:1	Test1	0.708	0.262	0.37
	Test2	0.717	0.258	0.36
**Lipid Partitioning**				
SA:GMS		PC lipid/water		
1:0		0.24		
0:1		0.14		
1:1		0.18		
2:1		0.26		
3:1		0.12		
1:2		0.16		
1:3		0.11		

*NA= Nicotinamide, SA=Stearic acid, GMS= Glyceryl monostearate, PC=Partition coefficient.

#### Preliminary tests 

The first attempt for SLN preparation was based on the M. Gobbi et al. report; i.e. 0.35 mM SA, 0.07 mM phospholipon^®^ 90G, and 0.037 mM sodium taurocholate using mechanical stirring (700-900 rpm).^28^ Unfortunately, the reported particle size (45.7 ± 3 nm) could not be achieved.

To attain the proper particle size, different situations were explored. Differing values of SA, phospholipon^®^90G, and sodium taurocholate, and also the rate of stirring, were evaluated. All experiments were carried out in triplicate. Table 2 shows the different conditions which were experimented. The suitable level of the corresponding factors were determined by these preliminary tryouts and used for designing the D-optimal test.

#### D-optimal design 

Response surface D-optimal design was used to optimize the best experimental situation for SLN preparation. Stearic acid to Phospholipon^®^ 90G ratio (SA/Phos), nicotinamide to sodium taurocholate ratio (NA/T), and mixing apparatus were considered as independent variables. The levels of these factors were determined through preliminary tests. SA/Phos ratio of 5 to 7.5, and NA/T ratio of 10 to 15 were evaluated. While oil phase was added slowly to water phase, the effect of different mixing instruments - including stirrer (1200 rpm), bath sonication, and probe sonication- were studied on the characteristics of the prepared formulation. The optimum formulation was selected regarding the acquired particle size, size SPAN, zeta potential, and encapsulation efficiency.

The equations of the most accurate model were achieved and the best model that fitted the data was assessed. Design-Expert statistical software (version7, Stat-Ease Inc.) was used for this evaluation.

Outliers were determined using normal probability plot and Cook’s distance. The suitability of the model was demonstrated using lack of fit and plot of the residuals versus predicted values. Response surfaces plots were studied to demonstrate the optimum condition of evaluated variables.

### Characterization of SLNs 

#### Size, size SPAN and zeta potential of SLNs

The obtained SLNs were well dispersed in deionized water and their particle size based on the number diameter was measured by laser diffraction technique using particle size analyzer (Shimadzu SALD-2101, Japan).

**Table 2:** The effect of different variables on particle size and size SPAN (preliminary tests)

**Independent variables**					**Dependent variables**	
**Stearic acid (mM)**	**Phospholipon** ^®^ **90G(mM)**	**Sodium taurocholate (mM)**	**Mixing**	**Addition method**	**Particle size (µm)**	**Size SPAN**
0.35	0.07	0.037	700rpm	Pour	7.1	4.62
0.35	0.07	0.037	900rpm	Pour	4.06	4.66
0.35	0.07	0.037	1200rpm	Pour	1.83	2.2
0.35	0.07	0.037	Bath sonic.^*^	Pour	1.88	3.87
0.35	0.07	0.037	Probe sonic.	Pour	0.705	1.95
0.5	0.07	0.037	1200rpm	Pour	0.806	3.01
0.5	0.07	0.037	Bath sonic.	Pour	0.91	2.48
0.5	0.07	0.037	Probe sonic.	Pour	0.64	0.89
0.75	0.07	0.037	Probe sonic.	Pour	12.83	6.37
1	0.07	0.037	Probe sonic.	Pour	Aggregation occurred	
0.5	0.07	0.025	Probe sonic.	Pour	9.53	3.19
0.5	0.07	0.03	Probe sonic.	Pour	0.38	0.77
0.5	0.07	0.045	Probe sonic.	Pour	1.96	2.05
0.5	0.07	0.037	1200rpm	Slowly added	0.52	0.375
0.5	0.07	0.037	Bath sonic.	Slowly added	0.59	0.59
0.5	0.07	0.037	Probe sonic.	Slowly added	0.23	0.41
0.5	0.07	0.037	1200rpm	injected	0.641	0.53
0.5	0.07	0.037	Bath sonic.	injected	1.47	0.717
0.5	0.07	0.037	Probe sonic.	injected	0.45	0.501

* Sonication

To evaluate the size dispersity of SLNs, SPAN value was measured using the diameters of 90%, 50% and 10% of SLNs, obtained by laser diffraction technique (Shimadzu SALD-2101, Japan), as indicated in the following formula:

$$SPAN=\;\frac{d\left(0.9\right)-d\left(0.1\right)}{d\left(0.5\right)}$$

The SLNs were dispersed in deionized water and their zeta potential were determined using NANO-flex^®^ (Microtrac, USA) zeta potential analyzer.

#### Encapsulation Efficiency 

Diethyl ether was added to the lyophilized SLNs and mixed for 10 minutes to dissolve SA. NA was dissolved by addition of deionized water. The mixture was aggressively vortexed and centrifuged. The NA amount was assayed from the supernatant sample. The encapsulation efficiency (EE) was calculated using the following equation:

$$\%EE=\;\frac{Amount\;of\;Loaded\;Drug}{Total\;amount\;of\;Drug}\times 100$$

NA assay was performed using high performance liquid chromatography (HPLC) method (A20 Shimadzu, Japan). The system was consisted of analytical column of HRC-ODS (150×6.0mm), a pump-controller unit (A20 Shimadzu) and an injector that were equipped with 20µl loop. Mixture of phosphate buffer (pH=4.5), methanol and glacial acetic acid was used as the mobile phase with ratio of 63:32:5 v/v respectively. Flow rate was adjusted to 1.5 ml per minute. The UV detector’s wavelength was set to 254 nm. Chromatograms was analyzed using Solution Software. NA standard solution was used for method development. Validation tests were performed completely on the developed method. All tests were done in triplicate.

#### Particle morphology 

The morphology of the selected NA-loaded and blank formulations were characterized by scanning electron microscopy, SEM (Cambridge, S-360, U.K.). Samples were coated with gold using sputter coater (Fisons, model 7640, UK) and then captured.

#### In-vitro drug release 

In vitro release of NA from SLNs were studied using dialysis bag diffusion technique. The selected formulation was placed in dialysis tube and suspended in continuously stirred medium of freshly prepared phosphate buffer, PBS (pH 7.4) and incubated at 37 °C (JAL Tajhiz lab tech, JTSL 20, Iran). Samples (2 ml) were withdrawn at different time intervals (at the beginning of test, 5, 15 and 30 minutes and 1, 2, 4 and 6 hours) and replaced with the same volume of fresh PBS. The withdrawn samples were analyzed by HPLC and their NA amounts were calculated. All measurements were done in triplicates.

The NA release profile was plotted in zero-order, first-order and Higuchi models. The best fitted model was determined using the acquired linear regressions.^29^

#### In-vitro cytotoxicity study 

Cytotoxicity assay was performed on SH-SY5Y human neuroblastoma cells using MTT cell viability test. Cells were cultivated in Dulbecco’s modified Eagle’s medium (DMEM) that were supplemented with 10% fetal bovine serum and 1% penicillin-streptomycin.

Cells were seeded in 96-well plates at concentration of 1×10^4^ cells per well. The NA solution, and suspensions of the blank SLNs, and NA-loaded SLNs (150 µl) were incubated with cells. After 24 h incubation, the content of each well was replaced with the same volume of media containing MTT (5 mg/ml). Plates were incubated for another 4hours, then MTT was removed and replaced with 150 µl of DMSO. Using microplate reader (Anthos 2020, USA), cell viability was assessed at 570 nm. Control cells were assumed to have 100% viability.

### Data analysis and statistics

The statistical model design was conducted using Design-Expert statistical software (version7, Stat-Ease Inc.). For other statistical analysis, ANOVA test were performed by SPSS^®^ statistics 17.0 (windows based version). Statistical significant was considered to be *p* value <0.05.

## Results

### Pre-formulation studies 

Linear correlation was found between UV absorption and NA concentration in water (1-100 mg/ml) and in n-octanol (0.1-5 mg/ml). NA solubility in water and n-octanol are reported in Table 1. Table 1 also demonstrates the calculated partition coefficient of duplicate runs of three different solvent ratios. The average partition coefficient was 0.42 ± 0.05. Negative LogP value of -0.37 confirms that NA is a naturally hydrophilic substance. The partitioning of NA in SA and GMS at different ratios is also reported in Table 1. Partitioning of NA in SA and also in the mixture of SA and GMS at the ratio of 2:1 was greater than at the other investigated ratios (Pc calculated to be 0.24 ± 0.17 and 0.26 ± 0.2, respectively).

**Figure 1:**
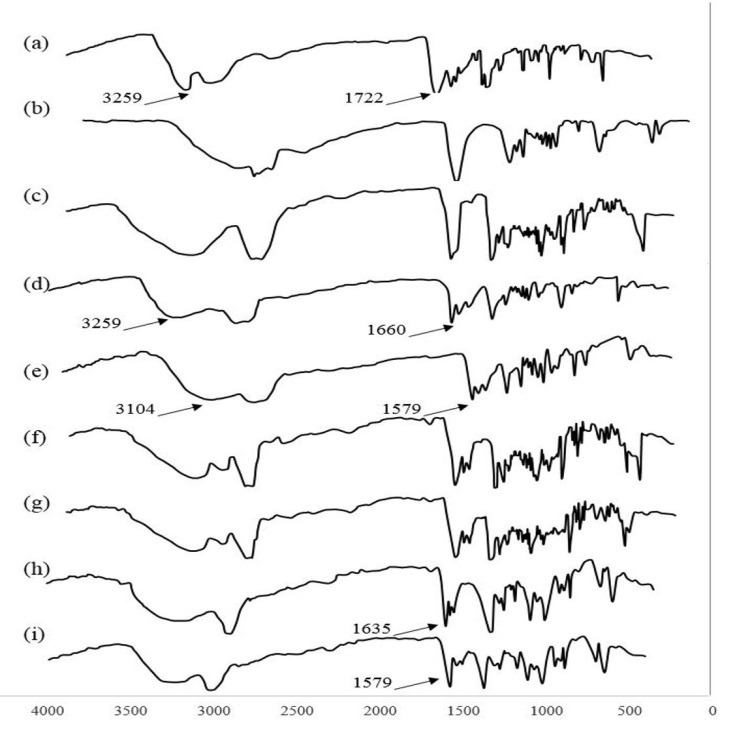
FTIR spectrums of (a) Nicotinamide, (b) Stearic acid, (c) Glyceryl monostearate, (d) physical mixture of Nicotinamide - Stearic acid, (e) melted-cooled sample of Nicotinamide - Stearic acid, (f) physical mixture of Nicotinamide - Glyceryl monostearate, (g) melted-cooled sample of Nicotinamide - Glyceryl monostearate, (h) physical mixture of Nicotinamide - Stearic acid - Glyceryl monostearate, and (i) melted-cooled sample of Nicotinamide - Stearic acid - Glyceryl monostearate.

The FTIR spectrum of NA and lipid(s) are shown in Figure 1. The -NH stretch peak of NA was shifted from 3259.1 cm^-1^ to 3104.8 cm^-1^ in NA-SA melted-cooled samples. No shift was observed for this bond at NA-SA physical mixture spectrum. The -C=O stretch peak was shifted from 1722.1 cm^-1^ in NA to 1660.4 cm^-1^ in NA-SA physical mixture and to 1579.4 cm^-1^ in NA-SA melted-cooled samples. No significant shifts were observed in either the NA-GMS physical mixture or their melted-cooled samples. The -C=O stretch peak was also shifted to 1635.5 cm^-1^ in NA-SA-GMS physical mixture, and to 1579.4 cm^-1^ in NA-SA-GMS in melted-cooled samples.

**Figure 2:**
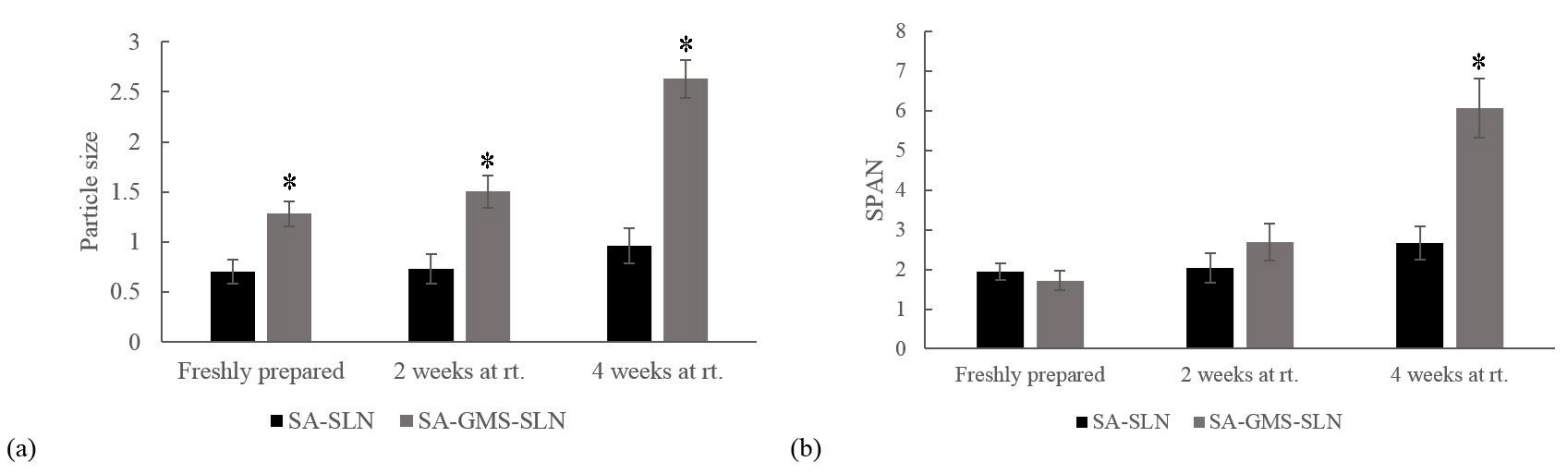
Characterization of SLNs prepared by stearic acid (SA-SLN) and mixture of stearic acid and Glyceryl monostearate with 2:1 ratio (SA-GMS-SLN). (a) Particle size and (b) size SPAN of freshly prepared particles and particles kept at room temperature for 2 and 4 weeks. (* *p* value <0.05).

Particle size, size SPAN and stability of the acquired particles with SA and also the mixture of SA and GMS (2:1), are reported in Figure 2. Using SA alone, stable nano-sized particles were achieved.

### Preparation of SLNs 

#### Preliminary tests 

As mentioned previously, SLNs were first prepared using 0.35 mM SA, 0.070 mM phospholipon^®^ 90G, 0.037 mM sodium taurocholate and mechanical stirring of 700-900 rpm. Unlike the reported data (45.7 ± 3 nm),^28^ the particle size in the micrometer range, i.e. 7.1 ± 4.62 µm and 4.06 ± 4.66 µm were obtained using the aforementioned stirring rates respectively (particle size was obtained before filtration).

To determine the most effective parameters affecting particle size, various factors such as mixing intensity, the amount of SA and sodium taurocholate, and the method of organic phase to the aqueous phase addition were investigated. Table 2 summarizes these results. The smallest particle size of 230 nm with SPAN value of 0.41 was acquired using the 0.5 mM SA, 0.07 mM phospholipon^®^ 90G, and 0.037 mM sodium taurocholate while slowly adding the oil to water phase under probe sonication.

The appropriate range of SA, phospholipon^®^ 90G, or sodium taurocholate concentrations was determined for further evaluation on the basis of these results.

#### D-optimal design

**Figure 3:**
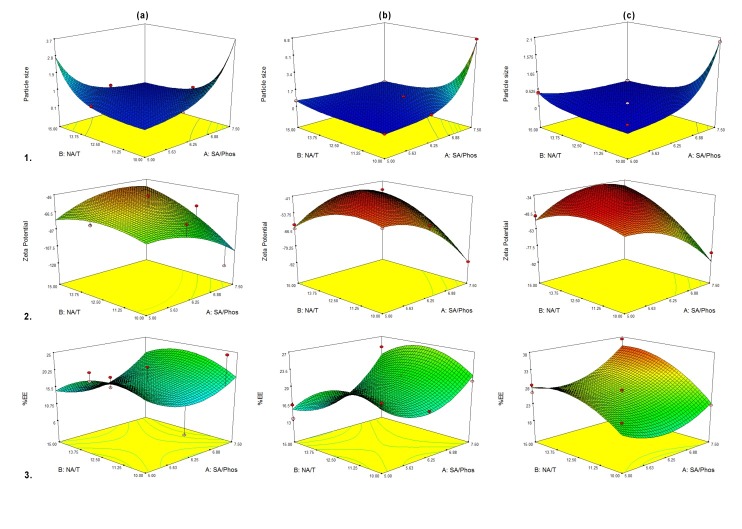
Response surface plots. Effect of Stearic acid to Phospholipon^®^ 90G ratio (SA/Phos) and nicotinamide to sodium taurocholate ratio (NA/T) on (1) particle size, (2) zeta potential and (3) encapsulation efficiency (%EE) in different mixing situation of (a) stirrer, (b) bath sonication and (c) probe sonication.

According to the preliminary tests, independent variables of SA/Phos ratio, NA/T ratio and mixing apparatus; and dependent responses of particle size, zeta potential and encapsulation efficiency were chosen for optimization studies. Design and results of the D-optimal experiments are shown in Table 3. All the responses were polynomial and fitted to the quadratic model with no transformation except for the particle size, which transformed in base 10 log. Table 4 shows the analysis of variance for the models. These models were considered significant since their *p*-values are <0.05. The effect of these independent variables on corresponding responses is plotted in Figure 3.

**Table 3:** The dependent and Independent variables, experimental design matrix and results of D-optimal design.

**Run**	**Independent variables**			**Dependent variables**			
	**A**	**B**	**C**	**Y1**	**Y2**	**Y3**	**Y4**
	**SA/Phos** **In range** ^a^	**NA/T** **In range** ^a^	**Mixing** **In range** ^a^	**Particle size** ^*^ **Minimize** ^a^	**Size SPAN**	**Zeta potential** **In range** ^a^	**%EE** **Maximize** ^a^
1	6.80	12.50	bath	0.469	0.59	- 54.5	20.65
2	5.00	12.92	stirrer	0.63	3.61	- 71.2	19.54
3	5.00	12.92	stirrer	0.662	3.65	- 71.8	21.87
4	5.00	15.00	probe	0.389	1.59	- 51.5	26.43
5	6.45	15.00	stirrer	0.529	0.64	- 55.6	11.91
6	5.00	10.00	bath	0.07	0.73	- 49.1	21.59
7	7.19	10.00	stirrer	-	2.33	- 127.5	24.99
8	5.00	15.00	probe	0.435	0.55	- 51.1	28.66
9	6.03	10.00	stirrer	0.57	2.31	- 65.7	6.44
10	7.50	10.00	probe	1.976	3.76	- 83.6	22.68
11	6.25	10.00	bath	0.495	0.54	- 55.3	17.54
12	5.00	15.00	bath	0.577	1.35	- 65.5	16.29
13	6.25	12.50	probe	0.088	0.74	- 46.2	27.17
14	7.50	12.08	stirrer	0.608	4.19	- 68.1	17.05
15	5.31	10.63	probe	0.068	0.69	- 49.8	23.12
16	7.50	15.00	probe	0.112	0.69	- 41.5	37.24
17	6.25	12.39	stirrer	0.162	4.21	- 46.9	21.20
18	7.50	10.00	bath	6.701	0.64	- 91.4	21.22
19	6.45	15.00	stirrer	0.494	2.19	- 55.1	14.98
20	7.50	15.00	probe	0.095	0.75	- 40.3	33.41
21	7.50	15.00	bath	0.231	0.37	- 48.2	24.65
22	5.00	15.00	bath	0.547	0.83	- 62.4	13.40

SA/Phos (stearic acid to Phospholipon^®^90G ratio); NA/T (nicotinamide to sodium taurocholate ratio); %EE=%Encapsulation efficiency. * reported in µm ^a^ Constrains

**Table 4:** Analysis of variance for D-optimal refined models and the suggested independent variables for optimum preparation condition.

**D-optimal design**	**Source of variation**	**Sum of Squares**	**Degree of freedom**	**Mean Square**	**F value**	**P>F**	
Particle size	Model	4.55	9	0.41	32.14	<0.0001	Significant
	Residual	0.14	11	0.015			
	Pure Error	4.676E-003	5	9.352E-004			
Zeta potential	Model	6760.32	11	614.57	4.42	0.0132	Significant
	Residual	1391.86	10	139.19			
	Pure Error	5.91	5	1.18			
Entrapment efficiency	Model	806.01	11	73.27	3.46	0.0303	Significant
	Residual	212.00	10	21.20			
	Pure Error	21.47	5	4.29			
**Suggested suituation**							
				**Observed**	**Predicted**	**% Observed to predicted ratio**	
**SA/Phos= 7.5**			size	0.107	0.103	103.819	
**NA/T= 14.74**			Zeta Potential	-40.90	-40.30	101.489	
**Mixing: Probe sonicaton**			%EE	36.026	35.277	102.123	

SA/Phos (stearic acid to Phospholipon^®^90Gratio); NA/T (nicotinamide to sodium taurocholate ratio)

#### Validation 

The final equations in terms of coded factors were:

**Log10(Particle size)=** -0.75 +0.21× A -0.093× B +0.25× C[1] +0.039× C [2] -0.56× AB -0.24× AC[1] +0.21 ×AC [2] +0.15 ×A2 +0.29 ×B2

**Zeta Potential=** -46.98 -6.01 ×A +10.83 ×B -12.36 ×C[1] +3.74 ×C [2] +14.23 ×AB +1.74 ×AC[1] -1.35 ×AC [2] +3.72 ×BC[1] -4.50 ×BC [2] -11.42 ×A2 -9.04 ×B2

**%EE=** +21.17 +2.01 ×A +0.96 ×B -3.84 ×C[1] -1.47 ×C [2] +1.42 ×AB -0.58 ×AC[1] +0.39 ×AC [2] -1.45 ×BC[1] -2.04 ×BC [2] +4.31 ×A2 -3.69 ×B2

**Figure 4:**
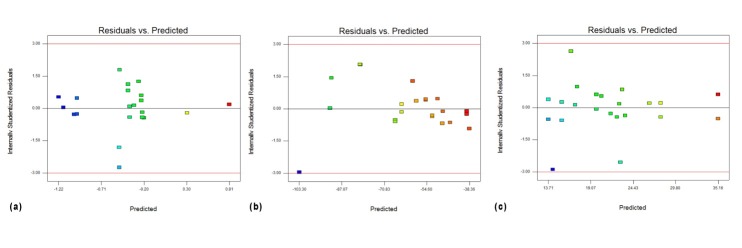
Residuals Vs. predicted graphs of (a) Particle size (b) zeta potential and (c) encapsulation efficiency.

To be able to use the acquired equations confidently, validation tests were performed. It can be concluded from these results that the model can be fitted despite some contradictory data. The best situation according the available data was achieved (Table 4). Plot of residuals versus predicted values demonstrates the suitability of the models (Figure 4).

### Characterization of selected SLNs

#### Size, size SPAN and zeta potential of SLNs

The optimum preparation situation, i.e. SA/Phos ratio of 7.5 and NA/T ratio of 14.74 using probe sonication, was used to prepare blank SLNs and NA-loaded SLNs. Blank SLNs with size of 101 nm and zeta potential of -37.5 were acquired. NA-loaded SLNs of 107 nm with zeta potential of -40.9 were achieved.

#### Encapsulation Efficiency 

NA assay was performed using the validated calibration curve (y=0.1385x + 0.0491 and r^2^ 0.9972) with acceptable precision and accuracy. In the optimum preparation condition, the encapsulation efficiency of 36% was achieved (Table 4).

#### Particle morphology 

**Figure 5:**
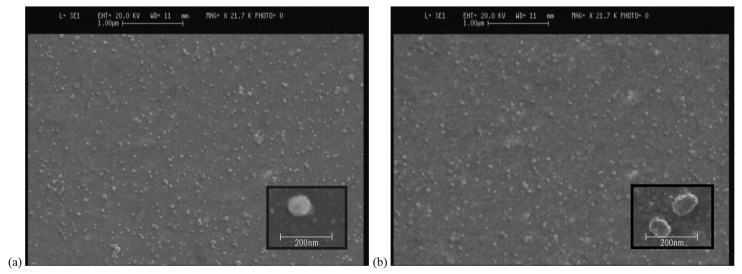
Scanning electron microscopy of the (a) Blank SLNs and (b) NA-loaded SLNs prepared in optimum condition; i.e. stearic acid to Phospholipon^®^ 90G ratio (SA/Phos) of 7.5 and nicotinamide to sodium taurocholate ratio (NA/T) of 14.74 using probe sonication.

The final formulations were evaluated in respect of their morphologies (Figure 5). The SEM shows almost uni-dispersed and spherical particles with agreeable sizes that were obtained by laser diffraction technique.

#### In-vitro drug release 

**Figure 6:**
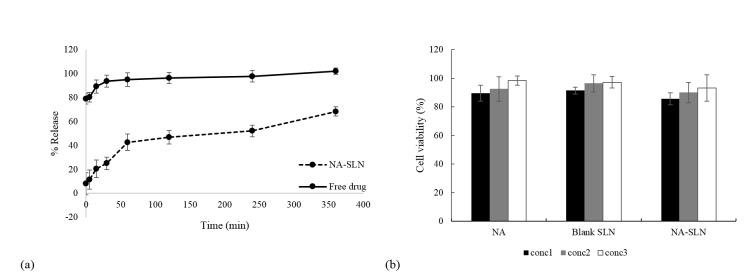
(a) The release profile of nicotinamide from SLNs (NA-SLN) and free drug; (b) Cytotoxicity evaluation of nicotinamide (NA), blank SLNs and NA-loaded SLNs (NA-SLN). Concentration 1, 2 and 3 represent the equivalent amounts of 120, 60 and 30mg NA (calculated based on the loading parameters).

The release profile of NA from the SLNs was studied (Figure 6). Approximately 50% of the drug was released in 4 h. It best fitted the Higuchi model, acquiring the highest r^2^ value.

#### In-vitro cytotoxicity study 

Figure 6 also demonstrates cytotoxicity of the acquired particles. According to the drug loading parameters, the amount of SLNs was calculated to represent NA concentrations of 120, 60, and 30 mg. The data shows that neither the NA nor the SLNs were toxic to the cells in the evaluated concentrations.

## Discussion

Biocompatible nano-particulate systems improve the controlled delivery of hydrophilic drugs to their sites of action, increase the therapeutic effect, and decrease the frequency of administration and thus the side effects of the drug. However, the low encapsulation efficiency of hydrophilic drugs in SLNs is a challenge in preparation of these pharmaceutical formulations. Through conducting proper pre-formulation studies and using statistical approaches such as D-optimal response surface tests, particulate systems with appropriate particle size, zeta potential, and encapsulation efficiency can be achieved. The present study aimed to achieve SLNs with efficient encapsulation of water soluble drugs possessing acceptable characteristics for sustained delivery.

NA is an HDAC inhibitor that could disrupt the progression of neurodegenerative disorders, like Alzheimer’s disease. It could also play an important role in preserving the integrity of mitochondria by evolving in the electron transport chain. As a result, it could reduce the vulnerability of neurons to oxidative stresses.^30^ Developing the high encapsulated system of such a freely soluble drug could benefit its delivery besides giving hope for the development of delivery systems for other soluble drugs.

The aqueous solubility of the drug directly affects its encapsulation efficiency into the SLNs. The solubility of NA in aqueous solvent ranges from 691 to 1000 mg/ml.^11^ In the present investigation, NA had a solubility range of 735.1 mg/ml in water (Table 1). The acquired negative logP of -0.37 confirms that NA is a naturally hydrophilic substance. The variation of acquired logP at different ratios of water to n-octanol was within the reported acceptable limits of ± 0.3.^9^

The more the drug partitioned in the lipid(s), the higher was the encapsulation efficiency achieved. Therefore, the effects of partitioning of NA in SA, GMS, or their mixture at different ratios were investigated. Best partitioning was acquired in the mixture of SA to GMS with the ratio of 2:1. However, primary studies demonstrate that the particles obtained from this mixture are not stable in time (Figure 2). Hence, the second best lipid with high amount of NA partitioning, SA, was investigated instead. A stable particulate system with smaller particle size was achieved (0.7 µm in comparison to 1.3 µm in mixture of SA to GMS with ratio of 2:1).

Any interaction between the drug and the lipid lowers the affinity of the drug for leaving the primary microemulsion and, therefore, the SLNs. This improves the loading parameters.

The warmed primary microemulsion was poured into cold water to quench and solidify the particles during the preparation process. Considering this, the mixture of drug and lipid(s) was melted and then cooled to evaluate the solubility and possible interaction of the drug with lipids in the preparation process. The interaction was evaluated using FTIR studies.

The interactions between the functional groups were indicated through observation of shifting in the corresponding stretch bonds in the FTIR spectrum of the final compound. There was no significant shift in the presence of GMS; however the FTIR spectra showed obvious shifting for both -NH and -C=O peaks in NA-SA and NA-SA-GMS melted-cooled samples.

According to the results of these pre-formulation tests, SA was chosen for the further investigations.

The microemulsification method often results in the preparation of SLNs with reproducible sizes. However, there could be many parameters affecting the results. Various experiments were conducted to determine the most effective parameters regarding the size and also size SPAN as the size distribution indicator (Table 2).

Mixing intensity was among the studied parameters. In this matter, magnetic stirrer with a stirring rate of 1200 rpm was evaluated; bath sonication and probe sonication were also studied as mixing apparatuses. According to these results, nanoparticles prepared by using magnetic stirrer with a stirring rate of 1200 rpm had the same size as nanoparticles prepared by using bath sonication. However, size SPAN expanded in the bath sonication method. The data showed that mixing intensity is an important factor affecting both responses.

Different amounts of SA were also studied. Large particles formed or aggregation occurred at high amount (>0.75 mM).

Another factor affecting the characteristics of the final nanoparticles is the method of adding the organic phase to the aqueous phase. The organic phase can be added to the aqueous phase through pouring, injecting, or slow addition. Although the injection tip was warmed to inhibit the solidification of the lipid phase, narrow tips were not suitable due to the viscosity of the lipid. On the other hand, using the wider tips for injection resulted in larger particles. Slowly adding seems to be a more suitable method than the others since this results in smaller particle size and narrower particle size distribution.

The most assumingly effective independent variables for adjusting the preparation condition of SLNs were chosen considering these preliminary studies. Basically, by increasing the number of independent variables, the number of experiments needed to substantiate also increased exponentially. In this study the ratio of parameters, i.e. SA/Phos ratio and NA/T ratio, were considered to reduce the number of independent variables and subsequently the number of trials.

Response surface D-optimal design is a proven statistical method that can be used to optimize the best experimental situation for preparation of SLNs. It results in mathematical models for multifactor experiments. One could estimate the effect of the studied factors for a desired response.

The independent variables of SA/Phos ratio, NA/T ratio, and mixing apparatus, and dependent responses of particle size, zeta potential, and encapsulation efficiency were chosen for the optimization studies.

The *p*-value was used to indicate the statistical significance of the models. The *p*-value lower than 0.05 indicated that the model is significant with 95% confidence. All responses were fitted into the quadratic model and all the quadratic models were significant according to ANOVA results (Table 4).

Particle sizes were transformed in base 10 log. The smallest particle size of 68 nm was achieved with SA/Phos ratio of 5.31 and NA/T ratio of 10.63 by using the probe sonication (Table 3). Quadratic model suggested. Table 4 shows the result of the ANOVA test for the measured particle size. The F value of the model implies that this quadratic model is significant. All the variable factors had a significant effect on particle size: however, mixing apparatus was found to be the most effective one. Normally closed amounts of Predicted R-Squared to Adjusted R-Squared were expected. The “Pred. R-Squared” of 0.8433 is in reasonable agreement with the “Adj. R-Squared” of 0.9334. Signal to noise ratio was indicated by Adequate Precision. The resultant ratio of 23.508 showed the adequate signal. The effects of the independent variables on particle size are plotted in Figure 3. It was concluded that whenever the stirrer was used, particle size would increase due to increase in the SA/Phos ratio or increase in the NA/T ratio. However, in cases where bath or probe sonication was used, increase in the SA/Phos ratio increased the particle size, although increase in the NA/T ratio did not significantly affect the particle size. This shows that mixing apparatus is the most effective parameter in this matter.

Table 4 also shows the results of the zeta potential modeling tests. No transformation was performed on the data. The 2FI model was suggested. However, the result of the ANOVA test showed that the quadratic model was also significant: this was preferable since it could exhibited the correlation in surface, and hence show the possible interaction between parameters. Although the mixing apparatus was significant in this model, NA/T ratio has the greatest influence. Further research should be undertaken to ensure that the model is fit. The effects of the independent variables on zeta potential are demonstrated in Figure 3. It can be concluded that no matter which apparatus is used for mixing, zeta potential will decrease due to increase in SA/Phos ratio or increase in the NA/T ratio. The only difference is the decreasing pattern.

No transformation was performed on encapsulation efficiency data. Linear model was suggested. For evaluating possible interactions between the parameters, data was fitted in the quadratic model. This was significant considering the results of ANOVA test (Table 4). The only significant term of this model is the mixing apparatus. Figure 3 demonstrates the effects of variables on encapsulation efficiency.

The results of the analytical tests were summed up in the final equations for every response. Positive and negative values show the synergistic and antagonistic effect on responses respectively. Each coefficient also represents the degree of effectiveness of that factor. These equations could be used to predict responses in imaginary situations.

In order to check the validity of models, validation test was performed. It can be concluded from the results that the model could be fitted despite contradictory data. The best situation according the available data seems to be the stearic acid to phospholipon^®^ ratio of 7.5 and the nicotinamide to sodium taurocholate ratio of 14.74, using probe sonication as the mixing apparatus. In this condition, particle size of about 107 nm, zeta potential of about -40.9, and encapsulation efficiency of 36.02% was achieved.

The acquired zeta potential is comparable to the previously prepared SLNs.^28^ Considering the solubility of NA, the acquired encapsulation efficiency seems to be suitable in comparison to the previously reported loading parameters of water soluble drugs.^2,10^

Considering the optimum situation for SLN preparation, final SLNs prepared and their morphological characteristics demonstrate the agreeable size with the acquired size by laser diffraction method. The particles were almost uni-dispersed and spherical (Figure 5).

The release profile of NA from the particles was also investigated. Considering the hydrophilicity of the drug, there was a primary fast rate of drug release, which could be attributed to the NA incorporated in the superficial lipid layer. In the studied formulation, almost 50% of the drug was released within the 4 h (Figure 6). The release rate then slowly continued for more than 10 h (data did not show), releasing the NA incorporated in the inner layer of particles. The recovery study shows that there is no interaction or barrier in the procedure that interferes in the detection of NA concentration.

It is often preferable that the delivery systems do not present cytotoxic effects. The cytotoxicity profile of NA, blank SLNs, and NA-loaded SLNs were evaluated. The results indicated that toxicity of NA and SLNs are negligible at investigated concentrations.

## Conclusion

The present investigation demonstrates the benefits of pre-formulation and statistical studies in optimization of SLNs preparation and in improving the loading parameters of hydrophilic drugs into such carriers. Using the D-optimal surface response test, the effect of various parameters on particle size, zeta potential, and %EE were evaluated. All the responses fitted to the quadratic model and all models were considered pharmaceutically suitable. Using the suggested optimum situation with stearic acid to phospholiponratio of 7.5 and nicotinamide to sodium taurocholate ratio of 14.74, and probe sonication as the mixing apparatus, 107 nm particles with zeta potential of about -40.9 and encapsulation efficiency of about 36% were obtained. Although the obtained particles had initial burst release, they could control the drug release afterwards. Their toxicity was considered negligible on the evaluated cell line. Overall, the acquired SLN system could be beneficial for delivery of nicotinamide. Besides, the suggested optimum situation for SLN preparation could be useful for designing the delivery systems for other hydrophilic drugs which is an ongoing work in our laboratory.

## Acknowledgments

This study is part of PhD thesis supported by Tehran University of Medical Sciences (TUMS); Grant no. 94-02-33-29374.

Authors would like to acknowledge Dr. Ali-Mohammad Tamaddon from School of Pharmacy and Research Center for Nanotechnology in Drug Delivery, Shiraz University of Medical Sciences for his consult and cooperation throughout the study.

## Ethical Issues

Not applicable.

## Conflict of Interest

The authors report no declarations of interest.
